# Asymmetrical Effect of Levodopa on the Neural Activity of Motor Regions in PD

**DOI:** 10.1371/journal.pone.0111600

**Published:** 2014-11-04

**Authors:** Kristina Martinu, Atsuko Nagano-Saito, Stuart Fogel, Oury Monchi

**Affiliations:** 1 Centre de Recherche de l′Institut Universitaire de Gériatrie de Montréal, Montreal, Quebec, Canada; 2 Brain & Mind Institute, Department of Psychology, Western University, London, Ontario, Canada; 3 Department of Radiology, Université de Montréal, Montreal, Quebec, Canada; INSERM/CNRS, France

## Abstract

Parkinson's disease (PD) is a neurodegenerative illness often characterized by asymmetrical symptoms. However, the reason for this asymmetry and the cerebral correlates underlying symptom asymmetry are still not well understood. Furthermore, the effects of levodopa on the cerebral correlates of disease asymmetry have not been investigated. In this study, right-handed PD patients performed self-initiated, externally triggered and repetitive control finger movements with both their right and left hands during functional magnetic resonance imaging (fMRI) to investigate asymmetrical effects of levodopa on the hemodynamic correlates of finger movements. Patients completed two experimental sessions OFF and ON medication after a minimum of 12 hours medication withdrawal. We compared the effect of levodopa on the neural activation patterns underlying the execution of both the more affected and less affected hand for self-initiated and externally triggered movements. Our results show that levodopa led to larger differences in cerebral activity for movements of the more affected, left side: there were significant differences in activity after levodopa administration in regions of the motor cortico-striatal network when patients performed self-initiated and externally triggered movements with their left hand. By contrast, when patients used their right hand, levodopa led to differences in cerebellar activity only. As our patients were affected more severely on their left side, we propose that levodopa may help provide additional dopaminergic input, improving movements for the more severely affected side. These results suggest that the impact of reduced dopamine in the cortico-striatal system and the action of levodopa is not symmetrical.

## Introduction

Parkinson's disease (PD) is a neurodegenerative disease whose cardinal symptoms are rigidity, tremor and bradykinesia. Symptoms often manifest more severely on one side of the body, and this lateralization persists throughout the duration of the disease [1], [2]. The underlying physiological and functional cerebral substrates of disease asymmetry in PD and their interaction with levodopa are poorly understood. Here we used self-initiated (SI) and externally-triggered (ET) movements during fMRI to investigate the effect of levodopa on the neural patterns underlying movements of asymmetrically affected hands.

The involvement of cortical regions and the basal ganglia in movement has long been documented [3]–[6]. For right handed individuals, movements of the left hand lead to greater increases in activity in motor areas than right hand movements [7], and cortical, subcortical and cerebellar task-related activity has been shown to decrease with automaticity [8]. Although patients with PD can also reach movement automaticity, they show greater increases in cortical and cerebellar activity than healthy controls when doing so [9]. We have previously shown that regions implicated in the motor cortico-striatal circuit (putamen, thalamus and premotor cortex) are involved in both SI and ET movements [10], [11]. More specifically, in young healthy adults, we have shown that activity of the putamen increases during repetitive control, ET and SI movements performed with the right hand. When using the left hand, however, activity of the putamen plateaued for both ET and SI movements. We suggested that the gradual involvement of the putamen during right hand movements is masked by the lack of proficiency of the non-dominant hand [10]. However, it remains to be investigated whether symptom asymmetry may impact task-related cerebral activation, and the response in the affected regions to dopamine therapy.

When dopamine levels are deficient, such as in PD, dopamine replacement therapy such as levodopa and apomorphine can restore motor-related activity [12]–[14]. We have previously shown that the effect of levodopa in PD leads to an increase in activity in the putamen whether movements are SI or ET [11]. We did not, however, investigate the differences in the effect of levodopa on the neural patterns linked to asymmetrically affected hands.

The goal of the present study was to examine the effect of levodopa on the neural activation patterns underlying asymmetrically affected left and right hand movements. We hypothesized that levodopa may lead to an increase in activity in the motor cortico-striatal network during more affected-side hand movements. Consistent with our previous work, we further hypothesized that this effect would be equivalent for both SI and ET movements (i.e., not task-specific). In our group of right-handed patients, we selected patients with more pronounced symptoms on the non-dominant side. We hypothesized that levodopa might selectively act on movements of the left hand, perhaps compensating for symptom severity. Understanding the interaction between levodopa and disease asymmetry would allow for new perspectives on levodopa mechanisms and subsequent research, and treatment of, asymmetrical symptoms.

## Methods

### Participants

Eleven right-handed patients diagnosed with PD [mean age 62.36±6.70 (SD), 6 men] and with higher symptom severity on the left side participated (Table 1). All PD participants met the UK brain bank criteria [15] for the diagnosis of idiopathic PD. Motor disability of patients with PD was mild to moderate severity, according to the Hoehn and Yahr staging criteria [2]. Patients presenting any other neurological or psychiatric disorder were excluded. Handedness was assessed with the Edinburgh Handedness Inventory (mean 90.8±14.9), early signs of dementia were assessed using the Montreal Cognitive Assessment (MoCA) [16] (mean OFF 27.2±1.7, ON 27.2±1.5), and symptoms of depression in all participants were measured using the Beck Depression Inventory II (BDI-II) (mean 9.9±7.5). At each session, motor symptoms were measured with the unified Parkinson's disease rating scale (UPDRS-III) (OFF: 31.55±4.9 and ON: 22.95±5.8). Left and right subsections were separated to give left and right UPDRS scores (OFF: 11.5L/9.5R and ON: 9.4L/6.6R). In addition to levodopa, some patients also regularly took other anti-parkinsonian drugs such as COMT inhibitors (n = 4), MAO-B inhibitors (n = 6), dopamine agonists (n = 3) and others (n = 3). Patients remained off these other medications for both the ON and OFF sessions.

**Table 1:** Demographics for the twelve patients with Parkinson's disease.

							MoCA	UPDRS scores		
Patient	G	Age	Dur.	Meds	BDI-II	Hand.	(On/Off)	Total (On/Off)	Left/Right (On)	Left/Right (Off)
1	F	69	2	1, 2	0	100	27/27	33.5/39.5	14/15	13/15
2	F	51	5	1, 4	12	58	27/27	18.5/31	7.5/5	11.5/7
3	M	67	2	1, 3	6	68.5	24/27	26/29	10/5.5	10.5/8
4	M	69	9	1, 4, 5	27	89	28/25	23/34.5	7/7.5	11/10.5
5	M	58	2	1, 2, 3	16	100	26/30	16.5/21.5	7/3.5	9/5
6	M	54	5	1, 2, 3, 5	6	83.3	30/30	25.5/36	12/6.5	16.5/11.5
7	M	68	3	1	6	100	27/28	26.5/27.5	9/8.5	9/7
8	F	62	5	1, 3, 4	12	100	29/28	19.5/31.5	8/5	14/10.5
9	F	68	11	1, 3, 5	15	100	26/27	25/31.5	8/7.5	9.5/10.5
10	F	65	1	1, 2	3	100	29/26	26/36	13/6.5	13/11
11	M	55	2	1, 3	6	100	26/28	12.5/29	7.5/2	9.5/8.5
Average (SD)		62.36 (6.7)	4 (3.2)		9.9 (7.5)	90.8 (14.9)	27.2/27.5 (1.7/1.5)	22.95/31.55 (5.8/4.9)	9.4/6.6 (2.5/3.4)	11.5/9.5 (2.4/2.7)

Dur., years since illness onset; Med., parkinsonian medication (1, levodopa; 2, COMT inhibitor; 3, MAO-B inhibitor; 4, dopamine agonist; 5, other); BDI., Hand., Edinburgh Handedness Inventory score.

### Ethics consent

All participants provided written informed consent, and the protocol was approved by the Research Ethics Committee of the Regroupement Neuroimagerie Québec, following the guidelines of the Tri-Council Policy Statement of Canada, the Civil Code of Quebec, the Declaration of Helsinki and the code of Nuremberg.

### Procedure

All patients came for two counterbalanced scanning sessions (one OFF medication, one ON levodopa), and were asked to withdraw from all anti-parkinsonian medications for a minimum of 12 hours prior to each appointment (see [11]). Participants remained off medications for the OFF session. For the ON session, participants took their usual dose of levodopa 1 hour prior to the beginning of MRI acquisitions. All participants practiced three blocks of the finger-movement task (for a total of 9 repetitions of each condition) prior to the scanning session to ensure they were comfortable performing it in the scanner.

### Task

Participants performed SI, ET and control (CTL) finger movements using left and right hands separately during functional MRI acquisitions in a pseudo-randomised order across runs, in both ON and OFF conditions (previously described in [11]). Each block began with written instructions, displayed for 2.5s, followed by the appearance of five squares oriented in a horizontal row on the screen, each corresponding to a button on the response box. Participants used all fingers except for the little finger; the square corresponding to the little finger was displayed for hand positioning, but remained inactive. The squares displayed on the screen turned green to indicate when a particular button should be pressed, and turned yellow for the duration of the button press. In the control condition, participants were instructed to repeatedly press a single button chosen at random for the duration of the block. In the ET condition, participants followed a randomly generated sequence. Finally, in the SI condition, participants generated their own sequences of finger movements. Participants were instructed to avoid pressing the same button consecutively in the SI task (this was considered an error), and to refrain from automatic (e.g., 1-2-3-4 or 4-3-2-1) or repeated sequences. For all tasks, participants were instructed to keep a comfortable, regular pace. Task conditions alternated at random after 20 button presses. An incorrect selection resulted in an error, and the corresponding square turned red to provide feedback.

### fMRI

#### Data acquisition

Participants were scanned using a 3T Siemens TIM Trio MRI scanner at the Functional Neuroimaging Unit (UNF) of the Centre de Recherche de l′Institut Universitaire de Gériatrie de Montréal (CRIUGM). Each scanning session (ON and OFF) comprised a T1-weighted three-dimensional volume acquisition (voxel size 1 mm^3^) for anatomical localization, followed by four T2*-weighted functional echoplanar acquisitions with blood oxygenation level-dependent (BOLD) contrast. Each run consisted of 146 frames of 43 slices (matrix size 128×128, voxel size 2.34×2.34×3 mm) acquired at a repetition time of 3.5 seconds.

#### Data analysis

Data analysis was performed using fmristat software and minctools [19] using a similar analysis strategy to our previous studies [10], [11], and was based on a linear model with correlated errors. After discarding the first three frames, all images were realigned to the fourth frame for motion correction and smoothed using a 6 mm full width half-maximum (FWHM) isotropic Gaussian kernel. The design matrix of the linear model was first convolved with a difference of two gamma hemodynamic response functions timed to coincide with the acquisition of each slice. The linear model was then re-estimated using least squares to produce estimates of effects and their standard errors. The resulting effect and standard effect images as well as anatomical images were spatially normalized using the ICBM152 atlas [17], [18]. In a second step, runs and subjects were analyzed using a mixed-effects linear model. A random-effects analysis was performed by first estimating the ratio of the random-effects variance to the fixed-effects variance, and then regularizing this ratio using spatial smoothing with a Gaussian filter. The amount of smoothing was chosen to achieve 100 effective degrees of freedom [20]. Within-session analyses (SI vs. CTL, ET vs. CTL, SI vs. ET) were performed by direct comparison using the effects and standard deviation images. Between-session analyses (ON vs. OFF) were performed by direct comparisons using the effects and standard deviation images of all participants in both drug conditions. All peaks at a significance of p<0.001 uncorrected with a cluster size >100 mm^3^ are reported in result tables.

## Results

### Clinical scores

Patients had significantly lower UPDRS scores ON than OFF (p = 0.005). Patients were more affected on the left side, as indicated by the sum of right side versus left side UPDRS scores (Table 1). Levodopa did not change the asymmetry of the patients' symptoms. There was no significant effect of session order and no statistical differences between MoCA scores ON and OFF. There were no significant correlations between MoCA or BDI-II scores and behavioral performance during the finger-movement tasks.

### Behavioral performance during scanning

#### Reaction times

The mean reaction times for SI, ET and CTL tasks ON and OFF for the left and right hand are reported in Table 2. A 3-way repeated measures ANOVA comparing drug condition (ON/OFF), hand (left/right) and task (SI/ET/CTL) revealed a 3-way interaction (p = 0.044). Paired-sample t-tests were used to investigate the effect of medication for the SI, ET and CTR tasks performed with the LH and RH hands. There were no statistical differences between ON and OFF sessions for either task (SI, ET or CTL) when performed with either the left or right hand. Additional paired t-tests were used to investigate the effect of hand used to perform the tasks for either task (SI, ET or CTL) when performed either ON or OFF medication. Surprisingly, patients ON medication had significantly longer reaction times for the left hand compared with the right hand in the CTL task (p = 0.031).

**Table 2:** Mean reaction time (SD) and percent errors for SI, ET and CTL movements for left and right hand movements of patients OFF and ON medication.

		Mean RT (SD) in ms			Errors (%)		
		SI	ET	CTL	SI	ET	CTL
OFF	LH	842 (127)	1110 (231)	723 (122)	3.66%	9.05%	0.46%
	RH	833 (174)	1108 (318)	726 (119)	3.01%	9.41%	0.79%
ON	LH	857 (108)	1123 (133)	784 (139)	2.44%	7.88%	1.49%
	RH	856 (148)	1137 (174)	744 (176)	2.66%	10.93%	1.44%

#### Errors

The same analysis strategy was used for errors. The percentages of errors in SI, ET and CTL tasks ON and OFF for the left and right hands are reported in Table 2. A 3-way repeated measures ANOVA comparing drug condition, hand and task revealed an effect of task (p = 0.026) with more errors in the ET condition overall, but no effect of drug condition, hand or any significant interactions.

### fMRI results

#### Self-initiated movements

**Figure 1:**
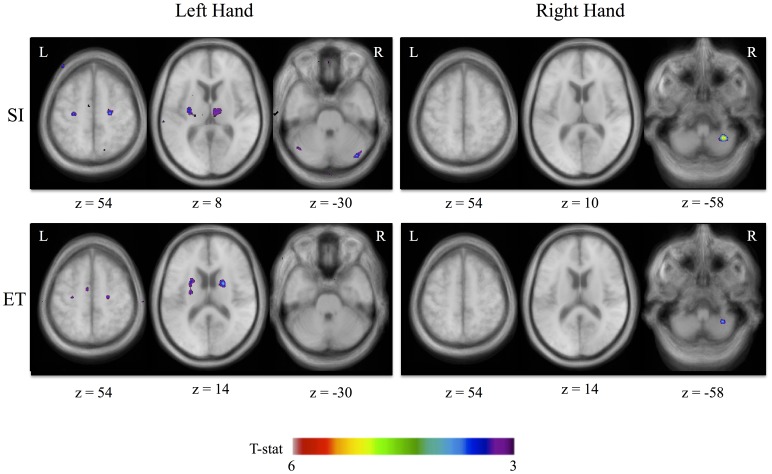
Brain activations for SI – CTL and ET – CTL movements for sessions ON vs. OFF for the more affected (left hand) and less affected (right hand) side. Anatomical images shown are the average of the T1 acquisitions of all patients transformed into stereotaxic space. The significant peaks are shown for t-stat values between 3 and 6.

When comparing patients ON and OFF levodopa administration (ON – OFF) for the SI – CTL contrast (Figure 1, Table 3), patients showed significantly increased activity in the right anterior prefrontal cortex (aPFC), left premotor cortex (PMC), bilateral motor cortex, and anterior cingulate cortex in the ON condition when using the left hand. Significant subcortical increases in activity were also observed in the left putamen, right thalamus and right cerebellum. For the right hand, patients ON showed significantly increased activity the right cerebellum only. There were no significant increases in activity in the OFF – ON comparison.

**Table 3:** Activation peaks between patients ON and OFF performing SI compared with CTL movements.

			Left hand					Right hand				
Anatomical area	BA	R/L	x	y	z	t	cluster	x	y	z	t	cluster
**ON> OFF**												
aPFC	46/10	R	44	48	18	3.46	112	-	-	-	-	-
PMC	6	L	−56	2	40	3.99	584	-	-	-	-	-
Motor	6	L	−28	−18	54	3.75	168	-	-	-	-	-
		R	24	−18	54	3.86	248	-	-	-	-	-
			24	−14	72	3.42	112	-	-	-	-	-
Cingulate	32	R	16	12	36	3.88	168	-	-	-	-	-
Putamen		L	−26	−12	18	3.52	600	-	-	-	-	-
			−28	−18	4	3.78	sc	-	-	-	-	-
Thalamus		R	12	−14	8	3.39	304	-	-	-	-	-
Cerebellum		R	40	−78	−28	3.85	344	30	−54	−58	5.46	648
**OFF> ON**												
-	-	-	-	-	-	-	-	-	-	-	-	-

The coordinates (x,y,z) in standard Montreal Neurological Institute stereotaxic space for all significant activation peaks for SI compared with CTL movements. Cluster sizes are in mm^3^.

BA, Brodmann area; R/L, right/left; aPFC, anterior prefrontal cortex; PMC, pre-motor cortex.

#### Externally triggered movements

For the ET vs. CTL contrast, patients ON showed significantly greater activity than OFF in the right motor cortex and left supplementary motor area (SMA), bilateral putamen and thalamus when using their left hand (Figure 1, Table 4). For the right hand, patients ON showed significant increases in activity in the right cerebellum. Compared to the ON condition (OFF – ON contrast), patients OFF had significant increases in activity in the left motor cortex when using their right hand, and no significant differences in activity for the left hand.

**Table 4:** Activation peaks between patients ON and OFF performing ET compared with CTL movements.

			Left hand					Right hand				
Anatomical area	BA	R/L	x	y	z	t	cluster	x	y	z	t	cluster
**ON> OFF**												
Motor	6	R	24	−18	54	3.44	64*	-	-	-	-	-
SMA	6	L	−6	−6	52	3.44	136	-	-	-	-	-
Putamen		L	−24	6	12	3.54	264	-	-	-	-	-
			−30	−16	8	3.60	256	-	-	-	-	-
			−32	−22	−4	3.47	104	-	-	-	-	-
		R	22	2	14	3.99	296	-	-	-	-	-
Thalamus		L	−12	−16	8	3.51	136	-	-	-	-	-
		R	8	−14	8	3.97	388	-	-	-	-	-
Cerebellum		R	-	-	-	-	-	30	−54	−58	3.89	240
**OFF> ON**												
Motor	6	L	-	-	-	-	-	-20	−8	68	4.51	320

The coordinates (x,y,z) in standard Montreal Neurological Institute stereotaxic space for all significant activation peaks for ET compared with CTL movements. Cluster sizes are in mm^3^.

BA, Brodmann area; R/L, right/left; PMC, pre-motor cortex; SMA, supplementary motor area.

## Discussion

The goal of this study was to examine the effect of levodopa on the neural patterns underlying asymmetrically affected hand movements in patients with PD. To our knowledge, this is the first direct evaluation of the impact of levodopa administration on behavioral and cerebral laterality. Surprisingly, although the literature suggests that PD patients tend to be more affected on their dominant side [21]–[22], the patients that participated in this study were mainly affected on the left, non-dominant side of the body. According to our results, levodopa had a preferential effect on brain activations in task-relevant brain areas during movements of the more affected hand. We found that regions involved in the motor cortico-striatal network (motor and pre-motor cortex, SMA, putamen and thalamus) and the cerebellum showed significant differences in activity between ON and OFF states when participants used their more affected (left) hand (Figure 1). In contrast, only the cerebellum showed significant differences ON vs. OFF when participants used their less affected (right) hand. Our results suggest that levodopa does not affect brain activity symmetrically, but rather, has a greater effect on the more affected side. There are two possible interpretations that are not necessarily mutually exclusive. First, as non-dominant hand movements are less automatic than dominant hand movements [7], levodopa may provide additional resources necessary to execute the less automatic left hand movements. Alternatively, levodopa may have a stronger effect on left hand movements because it was the most affected side in our patient cohort.

One existing theory may help to explain these results. According to the ‘focusing theory’, levodopa would help focus otherwise spatially diffuse activity [24] through an increase in signal-to-noise ratio by dopamine [25]. It is possible that dopamine facilitates activation of brain regions necessary for the generation of movements with the more affected limb. One positron emission tomography (PET) study in patients with PD at rest showed that levodopa decreased the PD-related pattern of brain metabolism by suppressing metabolic activity in the left motor cortex, putamen, right thalamus, and bilateral cerebellum [26]. Laterality effects were partially attributed to low statistical power; the authors speculated that the least affected side might have preferentially responded to levodopa because the nigrostriatal dopaminergic terminals of that side were less degenerated. Another group recently investigated the effect of a single dose of levodopa on a unimanual and bimanual grip task during fMRI in PD patients and healthy controls [27]. Levodopa significantly increased activity in the thalamus and putamen during bimanual movements. Although left side movements additionally recruited the ventrolateral thalamus, no significant differences between ON and OFF conditions were observed between the two hands. This may again be due to an effect of task. In summary, in accordance with the literature, our results suggest that there are differences in cerebral activity between the more and less affected sides.

One aspect worth mentioning is the lack of difference between reaction times in the ON and OFF states. On the contrary, when patients were ON medication, their reaction times for the control task were longer. It is important to note that the SI, ET and control tasks were not meant to test speed; participants were not instructed to perform as fast as possible. Rather, participants were asked to keep a steady, comfortable rhythm. For this reason, one possible explanation for the slower reaction times ON medication in the control task is that it is easier to keep a steady and regular pace than when OFF medication. In summary though, as expected, there were no significant differences in performance between ON and OFF conditions.

There seem to be considerable differences between the more and less affected hemispheres; asymmetrical degeneration of dopaminergic neurons in the substantia nigra underlies symptom asymmetry in PD [28]. It has been shown that the lateral ventricle contralateral to the more symptomatic side (i.e. the more affected hemisphere) is enlarged in PD patients with asymmetrical symptoms [29], and cognitive disruption often is consistent with the symptomatic hemisphere [30]. One study with *de novo* hemiparkinsonian patients looked that the effect of levodopa on cortical motor areas [31]. Patients showed a hypoactivity in motor regions contralateral to the more affected hand, reversible with levodopa, whereas the side contralateral to the unaffected hand showed almost constant activity. Furthermore, responses to levodopa have been shown to vary throughout the course of the disease. More specifically, responses tend to be mild and long-lasting in the early stages of PD, followed by greater responses with shorter duration times in the later stages, and ending in abrupt on and off switches [32]. The response to levodopa on the left and right side of the body varies depending on the patients' asymmetry, implying that the asymmetrically affected hemispheres represent different stages of the disease. Using four different finger tapping tasks, one study demonstrated that the more affected side showed reduced response latency, greater magnitude of improvement and shorter response duration to an infusion of levodopa [33]. In addition, another study showed the more affected side to have a delayed onset after oral levodopa administration [34]. Based on the timing of our MRI acquisitions (1 h after levodopa administration), it is possible that the effect observed between ON and OFF is related to differences in levodopa response of the more and less affected hemispheres. Hence, there are important differences between the more and the less affected hemispheres, such as the response to levodopa. This difference in latency may be involved in the differences in cerebral activity we observe during the more and less affected hand movements.

We have recently shown that greater recruitment of the putamen is necessary to compensate for a lack of automaticity to a greater extent for left hand rather than right hand movements [10] in a study using SI, ET and control movements in young healthy right-handed participants. Based on these previous results, we speculated that the increase in putamen activity reached a ceiling when participants used their left hand, whereas gradual increases in activity could be observed from control to ET to SI movements when using the right hand. In patients with PD performing the same tasks with their right hand only, we have previously shown that differences in putamen activity between control, ET and SI tasks were reduced compared with older healthy controls, and that levodopa led to non-task-specific increases in cortico-striatal activity [11]. This was in accordance with a study investigating arm-reaching movements during PET imaging that showed that levodopa increased motor task-related activity [12]. Taken together, our results suggest that levodopa increases cortical and subcortical activity in the left hand condition due to increased difficulty using the more affected hand irrespective of the task being performed. However this may be a combined effect of disease laterality and hand proficiency.

Although we cannot conclusively attribute the effect of levodopa to the hand used and/or disease asymmetry, our results have important implications for the mechanisms underlying levodopa function and the treatment of asymmetrical PD symptoms. Follow-up studies with a full cross-over design including left- and right-handed patients with left- and right-disease asymmetry will be necessary to further disentangle the relationship between levodopa's effect on movements as a factor of handedness and symptom and cerebral lateralization.
